# Mycetoma of the right foot: a rare clinical image

**DOI:** 10.11604/pamj.2022.42.273.35730

**Published:** 2022-08-11

**Authors:** Mohammed Kamran Shaikh, Sonali Borkar

**Affiliations:** 1 Department of Community Medicine, Datta Meghe Medical College, Wanadongri, Nagpur, Maharashtra, India

**Keywords:** Mycetoma, fungi, bacteria

## Image in medicine

Mycetoma is a disease caused by certain bacteria and fungi found in soil and water. These bacteria and fungi may enter the body through a break in the skin, often through the foot. We are presenting a case of a 42 years old male farmer, who comes to the emergency department with a complaint of swelling over on the foot. On inspection of the patient’s foot, there was swelling over the foot with the fungal inspection. Skin biopsy and laboratory investigation were suggestive of a mycetoma of the foot. On the confirmation, the patient was referred to the surgery department for further management.

**Figure 1:**
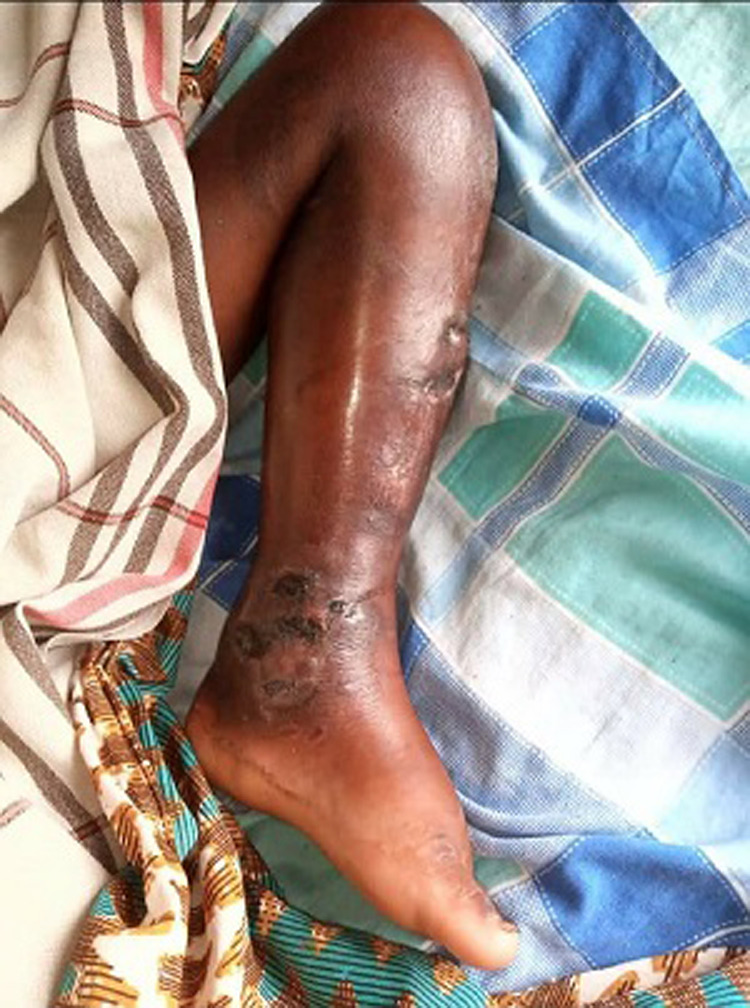
swelling over foot with fungal infection

